# Management of Inflammatory Root Resorption of Maxillary Central Incisors following Traumatic Avulsion

**DOI:** 10.1155/2021/6309711

**Published:** 2021-09-28

**Authors:** Seyed Mohsen Sadeghi, Samaneh Moradi, Samaneh Soltani

**Affiliations:** ^1^School of Dentistry, Department of Endodontics, Qom University of Medical Sciences, Qom, Iran; ^2^School of Dentistry, Department of Endodontics, Isfahan University of Medical Sciences, Isfahan, Iran; ^3^School of Dentistry, Department of Periodontology, Qom University of Medical Sciences, Qom, Iran

## Abstract

**Background:**

Avulsion is among the most severe types of dental trauma, which often occurs at young ages and can compromise the long-term prognosis of the traumatized tooth. *Case Report*. Herein, we report replantation of two avulsed teeth. Our patient was an 11-year-old boy with two avulsed maxillary central incisors due to a bicycle fall 2 months earlier. The patient was referred to us after rigid splinting of his teeth by a surgeon. Long-term calcium hydroxide (CH) therapy was performed for the patient, and after healing of periodontal ligament (PDL), apexification was performed for both teeth followed by root canal therapy. During the 2-year follow-up, both teeth were functional and had no radiographic or clinical evidence of resorption or ankylosis.

**Conclusion:**

The reported case highlights the favorably high tissue healing potential following severe dental trauma, given that appropriate treatment is performed. Correct endodontic management can guarantee the long-term prognosis of teeth following severe dental trauma.

## 1. Introduction

Severe dental traumas often cause complex dental fractures or injuries and can simultaneously involve the dental hard tissue, periodontium, and dental pulp. The currently used classification for dental traumas differentiates between the fracture injuries and tooth displacement/luxation. Concussion and subluxation are the most common displacement traumas while avulsion has a lower prevalence and accounts for 7% to 23% of dislocation traumas. Horizontal root fracture of permanent teeth is an uncommon incidence, with a prevalence rate of 0.5% to 0.7% [1]. The prevalence of avulsion is higher in children between 7 and 9 years, and often involves a maxillary incisor. Falls, fights, and sport injuries are often the main etiology [1]. The long-term consequences of dental trauma can adversely affect the long-term prognosis of the traumatized teeth and can even lead to tooth loss and its consequent adverse functional and esthetic sequelae. Thus, early management and regular follow-ups are imperative to prevent these consequences [2]. The level of injury to the periodontium of the avulsed tooth determines its prognosis. Thus, timely endodontic intervention of avulsed teeth is imperative to improve their long-term prognosis. The chance of pulpal regeneration in avulsed teeth is low (around 10%) even under ideal conditions. Therefore, there is high risk of complications if endodontic interventions are postponed or not performed [3].

The time interval between the avulsion and replantation of the tooth, as well as the storage medium in which the tooth is stored during this time period, are critical factors determining the periodontal prognosis of the avulsed tooth. Periodontal injuries due to dehydration or immersion in a nonphysiological solution would cause inflammatory or replacement/substitutive root resorption, which would highly jeopardize the success of subsequent treatments. If endodontic treatment is not performed or is performed after a delay, the root canal system becomes infected. The infection can reach the injured cementum through the dentinal tubules and cause infection-related root resorption [2]. In case of occurrence of inflammatory root resorption, endodontic treatment and induced formation of a root-end barrier (apical plug) by the application of calcium hydroxide (CH), mineral trioxide aggregate (MTA), or other biomaterials should be considered [4].

Treatment of horizontal root fracture is often limited to repositioning of the coronal segment and semirigid splinting for 2 to 4 weeks to provide function and comfort for the patient. Dental pulp often remains viable in 80% of the cases. In case of occurrence of the signs and symptoms of pulpitis, root canal treatment of the coronal segment is performed. In most cases, the apical segment remains vital and apical closure occurs in some cases [5].

This case report describes the management and 2-year follow-up of an 11-year-old boy with external root resorption of his avulsed maxillary left central incisor and horizontal root fracture and inflammatory root resorption of his avulsed maxillary right central incisor.

## 2. Case Report

### 2.1. Dental History

Our patient was an 11-year-old boy with a history of bicycle fall 2 months earlier, with the resultant dental trauma. He was taken to a hospital immediately after the accident, and his avulsed maxillary central incisors were replanted with a rigid splint (arch bar) by a surgeon after storage in milk for about an hour. The splints were removed by the surgeon after 1 month and the patient was referred to the Endodontics Department of the School of Dentistry.

### 2.2. First Treatment Session (2 Months after Trauma)

**Figure 1:**
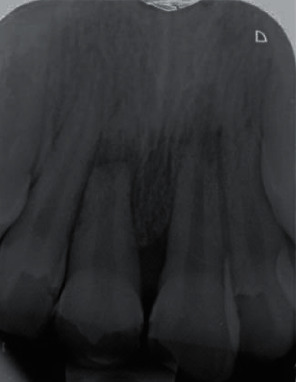
Periapical radiographs of the maxillary central incisors.

**Figure 2:**
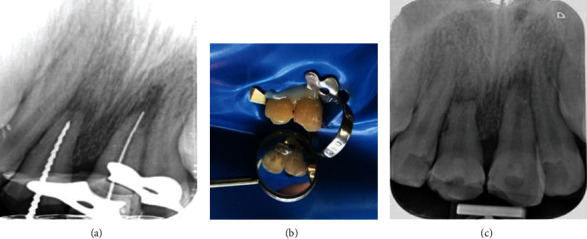
(a) Working length determination for the maxillary central incisors; (b) isolation with clamp, flowable composite, and wedge; (c) apexification with calcium hydroxide and temporary restoration of the access cavity.

Two months after the accident, the patient was referred to us for the first time. The patient was systemically healthy, and his facial soft tissue and gingiva were normal. Clinical and periapical radiographic examinations revealed horizontal root fracture in tooth #8 and external root resorption in teeth #8 and #9 (Figure 1). CBCT was needed, but the patient's parents did not accept due to radiation. The pulp vitality tests were performed. The traumatized teeth did not respond to the cold, heat, or electric pulp tests but were both sensitive to percussion and palpation and showed grade II mobility. Since 2 months had passed since the avulsion, long-term CH therapy was considered to stop the progression of inflammatory root resorption. Written informed consent was obtained from the patient and his parents. In the first treatment session, after local infiltration injection and access cavity preparation, canals were disinfected with 5.25% sodium hypochlorite, and filled with CH (Golchai, Iran) with creamy consistency. The teeth were then temporarily restored with Cavisol (Golchai, Iran) (Figure 2).

### 2.3. Second Treatment Session (1 Week Later)

**Figure 3:**
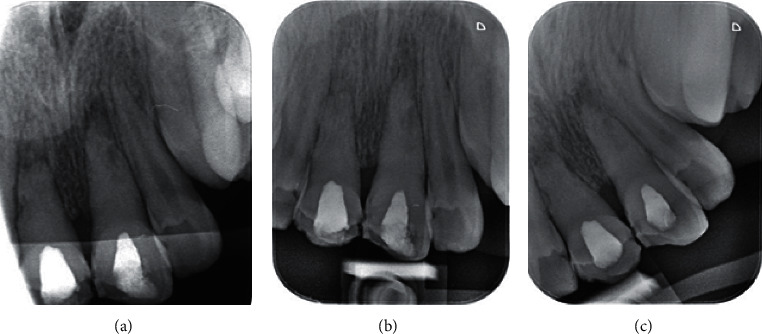
(a) Periapical radiograph taken at 1 month after the application of powdery calcium hydroxide; (b) periapical radiograph taken after 2 months; (c) periapical radiograph taken after 3 months, showing sound periodontal ligament.

The temporary restoration was removed; the root canals were rinsed with sodium hypochlorite, and filled with CH with powdery consistency. The teeth were followed-up monthly to accomplish the root canal treatment in case of observing a sound and intact periodontal ligament (PDL) and complete resolution of inflammatory root resorption (Figure 3).

### 2.4. Third Treatment Session (3 Months Later)

**Figure 4:**
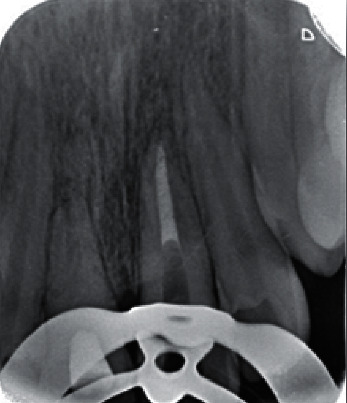
Periapical radiograph taken after one-session apexification of tooth #9 with MTA plug.

The process of root resorption had been stopped, and a sound PDL was observed on the radiographs. Considering the poor patient cooperation, we decided to accomplish the endodontic treatment of the teeth within two sessions. Since the apices were open (larger than a #60 K-file), a 4 mm MTA plug was applied at the root end, and then the coronal part of the canal was filled by a warm vertical condensation technique. Fast-setting MTA Angelus (Angelus, Brazil) was used for filling the apical part of the root canal. In tooth #9, the apical 4 mm of the apex was instrumented with hand K-files after elimination of CH, rinsed with sodium hypochlorite and saline, dried with paper points, and filled with MTA Angelus. Next, the coronal part of the canal was filled with gutta-percha and AH-26 sealer using a warm vertical condensation technique (Figure 4).

### 2.5. Fourth Treatment Session

**Figure 5:**
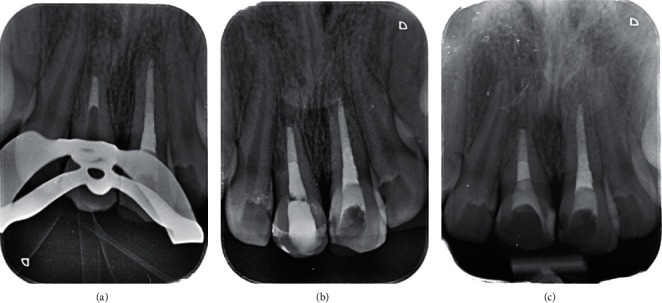
(a) Apexification of tooth #8 with MTA plug; (b) warm vertical root filling of teeth #8 and #9; (c) composite restoration of both teeth.

In tooth #8, the apical 4 mm of the canal was instrumented with hand K-files after elimination of CH, rinsed with sodium hypochlorite and saline, dried with paper points, and filled with MTA Angelus. The coronal part of the canal was then filled with gutta-percha and AH-26 sealer using a warm vertical condensation technique. The patient was then referred to the Operative Dentistry Department for restoration of both teeth (Figure 5).

### 2.6. Fifth Treatment Session (1 Year after Trauma)

**Figure 6:**
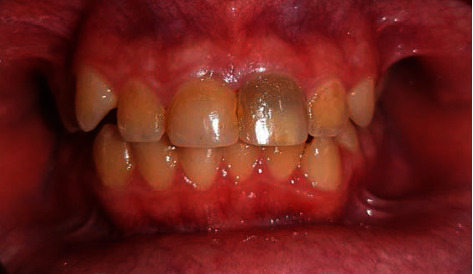
Discoloration of tooth #9 at the 1-year follow-up.

At the 1-year follow-up, tooth #9 showed discoloration (due to the bismuth oxide present in MTA Angelus) [6]. Thus, we decided to perform internal bleaching for correction of discoloration. The composite restoration of tooth #9 was removed, and gutta-percha was eliminated to 2 mm below the cementoenamel junction. A light-cured glass ionomer (GC, USA) was applied as a barrier, and sodium perborate was placed in the root canal as the bleaching agent. The tooth was then temporarily restored with the light-cured glass ionomer (Figure 6).

### 2.7. Sixth Treatment Session

One week later, the patient presented for continuation of bleaching treatment. Since the tooth color had been corrected, temporary restoration and sodium perborate were removed and the patient was referred to the Operative Dentistry Department for permanent restoration of the tooth.

### 2.8. Follow-Up (2 Years after Trauma)

**Figure 7:**
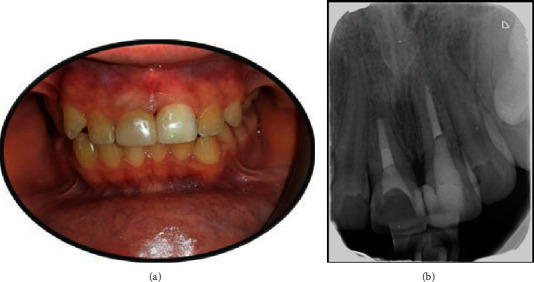
Teeth showed optimal function and esthetics at the 2-year follow-up.

The patient was completely satisfied with the function and esthetic appearance of his teeth. Both teeth were completely functional and had normal mobility. Radiographically, no periapical lesion was seen, and the inflammatory root resorption of both teeth had been completely resolved (Figure 7).

## 3. Discussion

Avulsion is a serious trauma to the dental pulp and PDL. Following avulsion and replantation, the tooth is at risk of infection and root resorption, which may compromise the prognosis of treatment and decrease the survival rate. Radiographic detection of root resorption and ankylosis is difficult shortly after replantation. Also, it is noteworthy that the risk of root resorption increases with time [7, 8]. Moreover, some cases with late complications following PDL healing can be falsely considered as healed if a follow-up session less than 1 year is scheduled [9].

The prevalence of dental trauma to permanent teeth is 25% to 33% [10], and crown fracture without pulp exposure is the most common type of dental trauma [11]. However, complex dental trauma as in our case rarely occurs. The study of complex and unusual cases of dental traumas can provide an insight into more efficient treatment of trauma cases.

In our patient, avulsed maxillary central incisors underwent rigid splinting by a surgeon after 1 h of storage in milk. Milk is a good storage medium considering its osmolarity and adequate pH and can preserve the PDL cells viable for up to 6 h [12]. Replanted teeth were clinically and radiographically followed-up for 2 years on a regular basis.

Current recommended clinical management of avulsed teeth includes intracanal CH placement initiated within 7–10 days after avulsion for a tooth with a closed apex to help prevent resorption. If endodontic treatment is started after this time period or after a resorptive root lesion is detected on a radiograph, CH is placed for an extended amount of time [13].

In our patient, since more than 2 weeks had passed since the avulsion, and the teeth showed external root resorption, long-term CH therapy was considered to stop the root resorption and then accomplish the root canal treatment. Radiographs were obtained on a monthly basis to assess the hard tissue formation and washout of CH, and also to accomplish endodontic treatment as soon as the root resorption stopped. Long-term treatment with CH may weaken the roots and make them susceptible to fracture. However, this effect was minimal in our case since CH was only used for 3 months [14].

Damage to the external root surface along with intracanal infection can cause inflammatory root resorption. Bacterial toxins originating from the root canal can reach the PDL through dentinal tubules and aggravate the external root resorption. Appropriate endodontic treatment can prevent the progression of resorption [15, 16].

The prognosis of horizontal root fracture depends on the site of fracture; the apical segment often survives while the coronal segment is necrotized in 25% of the cases. Also, fractures below the alveolar crest, as in our case (which was in the apical third of the root), often have good prognosis [5].

Apexification and revascularization have been considered effective treatment procedures for nonvital immature teeth. Apexification involves disinfection of the root canal, promoting an apical hard tissue barrier and obturating the empty canal space with root filling material. On the other hand, revascularization is aimed at promoting continued root development, the replacement of lost tissue, and function with stem cell-mediated growth of reparative tissue [17].

Endodontic treatment of immature teeth with horizontal root fracture is difficult due to the absence of an apical stop. In such teeth, one-session apexification with MTA can serve as an alternative to the traditional apexification with CH, and has shown promising results [18]. MTA can cause discoloration, and this property limits its application particularly in the anterior teeth [19]. According to recent studies, iron, manganese, and bismuth oxide are responsible for tooth discoloration. In fact, exposure of dentin collagen to bismuth oxide is responsible for tooth discoloration. Blood contamination is another cause of tooth discoloration. New bioceramic materials have the advantages of MTA without causing discoloration [20]. However, due to the unavailability of newer biomaterials in Iran and the need for fast-set MTA, MTA Angelus was used for our patient.

A rigid splint does not permit any physiological mobility of the tooth and thereby creates the conditions for complications in the sense of ankylosis or external resorption.

In the case of a semirigid splint, the physiological functional mobility of the traumatized tooth is possible, which is more favorable for the healing of PDL [21]. Semirigid splinting for 1 to 2 weeks is often recommended to prevent ankylosis. As most cases of avulsion occur in juvenile patients, it is of clinical importance to preserve the tooth and surrounding bone tissue to provide optimal, definitive, and prosthodontic rehabilitation under the possibility of later treatment with dental implants [22].

Splinting can be performed to immobilize the traumatized teeth whenever required. The prevalence of ankylosis after replantation of an avulsed tooth and rigid splinting is 57%-80%. Ankylosis in adults can be well managed by prosthetic and dental implant treatments. Rigid splinting is often contraindicated except for some certain cases of bone fracture, which can cause the death of PDL cells due to their compression, and can lead to subsequent root resorption. In our present case, due to the use of a rigid splint for 1 month, signs of inflammatory root resorption were noted at different parts of the root [23].

The follow-up radiographs of our case indicated healing of the PDL, and no further root resorption was noted. Several international surveys demonstrated the inadequate knowledge of dentists about emergency management of TDIs. As TDIs include other injuries and both GDPs and specialists in various dental disciplines can encounter such injuries in their practice, they must have sufficient knowledge on the emergency management of TDIs in general [24]. The current case report described successful management of maxillary central incisors following severe complex dental trauma.
